# Building a Hepatitis C Clinical Program: Strategies to Optimize Outcomes

**DOI:** 10.1007/s40506-018-0177-5

**Published:** 2018-10-18

**Authors:** Autumn Zuckerman, Alicia Carver, Cody A. Chastain

**Affiliations:** 1 Specialty Pharmacy Services, Vanderbilt University Medical Center, Nashville, TN 37232 USA; 2 Division of Infectious Diseases, Vanderbilt University Medical Center, A2200 MCN, 1161 21st Avenue, Nashville, TN 37232-2605 USA

**Keywords:** Care delivery, Cascade of care, HCV, Hepatitis C virus, Multidisciplinary care

## Abstract

**Purpose of review:**

An increasing number of specialists and non-specialists are developing clinical programs to treat and cure hepatitis C virus (HCV). The goal of this paper is to evaluate and describe optimal strategies to improve outcomes related to HCV care delivery.

**Recent findings:**

Screening and diagnosis of HCV should be guided by established recommendations. Given the recognized disparity in HCV diagnosis and linkage to care, a multi-modal approach involving care coordination and technology resources should be used to improve patient engagement. Access to HCV treatment may be optimized through systematic documentation, prior authorization, and appeal processes. Treatment monitoring should emphasize medication adherence, side effect and drug interaction management, as well as elimination of practical barriers. Finally, post-treatment engagement to promote liver health and reduce the risk of complications or reinfection maximizes the benefit of HCV treatment.

**Summary:**

The landscape of HCV treatment has evolved from a specialist-driven model with few patients qualifying for treatment to an opportunity for non-specialists and other providers to provide curative therapies in most patients. Innovative practice models that employ a multidisciplinary approach will likely improve screening, diagnosis, engagement, and treatment outcomes.

## Introduction

Direct-acting antiviral (DAA) treatments, with few side effects and high rates of sustained virologic response (SVR), have focused attention on new methods of care delivery to improve outcomes for patients with hepatitis C virus (HCV) infection. Establishing an HCV clinical program that is focused on optimizing the entire HCV cascade of care is necessary to effectively impact the epidemic and move towards HCV elimination. Outcomes of an HCV clinical program should be defined not only by the number of patients successfully cured but by successes in screening and diagnosis, linkage to care, accessing medications, completion of treatment, appropriate post-treatment monitoring, and reinfection risk reduction. With increasing literature about best practices, clinicians and programs can identify optimal strategies and opportunities to improve each step within this cascade. Here, we review these strategies and provide real-world insights to improve outcomes for the growing number of healthcare providers delivering care to patients with HCV. Table 1 summarizes recommendations to improve clinical practice at each step within HCV care delivery.

**Table 1:** Recommendations to improve outcomes when establishing an HCV practice

Practice element	Recommendation	Brief description
Screening and diagnosis	Guideline-recommended screening	All clients with known risk behaviors, risk exposures, or other pertinent medical conditions should be screened for HCV.
	Public health awareness	HCV clinical programs should promote community awareness of HCV and appropriate screening indications.
Linkage and access to care	Patient navigation program	Following a diagnosis of HCV, staff contacts patients for education and appointment scheduling. Specialists/patient navigators should monitor patients to ensure appointment attendance and follow-up by phone, in person, or other technology (app, portal, text, etc.) to engage those who miss an appointment.
	Telehealth	HCV providers utilize telecommunications such as an electronic portal, video conferencing, and telephone calls to engage patients in care beyond a clinic visit.
	Non-specialist care	Physicians, nurse practitioners, physician assistants, or pharmacists provide HCV evaluation and treatment exclusive of a gastroenterologist, hepatologist, or infectious diseases provider. Thorough education and training should be completed prior to prescribing HCV treatment.
Treatment access	Patient navigation program	Specialists/patient navigators should work closely with the patient and pharmacy to ensure all steps for medication approval are followed appropriately and to completion.
	Cost management	High out-of-pocket patient costs should be defrayed by specialist/patient navigator prior to therapy initiation.
Treatment monitoring	Patient navigation program	Specialists/patient navigators should work closely with the patient and pharmacy to ensure continued treatment access and appropriate follow-up. Linkage to additional support (i.e., mental health professional, social work) may be necessary to overcome barriers to treatment completion.
	Guideline-recommended monitoring	Patients should be monitored according to the AASLD/IDSA treatment guidance, focusing on continued assessment of adherence, drug-drug interactions, and adverse events while on treatment.
	Non-specialist care	Non-specialist providers provide HCV treatment monitoring. Thorough education and training of non-specialists should be completed prior to prescribing HCV treatment.
	Telehealth	HCV providers utilize telecommunications such as an electronic portal, video conferencing, and telephone calls to monitor patients while on treatment.
Post-treatment engagement	Patient education	All patients should be educated regarding the clinical implications for SVR, the risk of reinfection, and the need for further liver care depending on fibrosis status.
	Advanced liver disease care	All patients with advanced fibrosis should be linked to for liver disease care including hepatocellular carcinoma and esophageal varices screening.

*HCV* Hepatitis C virus, *AASLD* American Association for the Study of Liver Diseases, *IDSA* Infectious Diseases Society of America, *SVR* sustained virologic response

## Screening and diagnosis

The first step in the HCV cascade of care is screening and diagnosis. Historically, hepatitis C screening was recommended for those with specific risk behaviors, risk exposures, or specific medical conditions. These included, but were not limited to, injection drug use (IDU), percutaneous/parenteral exposures in unregulated settings as well as healthcare environments, blood component or organ transplant recipients prior to 1992, HIV infection, and those with unexplained chronic liver disease [1•]. One-time hepatitis C testing is now recommended by the US Preventative Task Force and Centers for Disease Control and Prevention for persons born between 1945 through 1965 to better identify at-risk persons without relying on risk factor-based screening [2, 3]. More recently, the American Association for the Study of Liver Diseases (AASLD)/Infectious Diseases Society of America (IDSA) HCV Guidance recommended screening all pregnant women for HCV [1•, 4•]. These recommendations have evolved to optimally screen the at-risk population and capture those who are actively infected with HCV based on epidemiology trends. Ongoing re-assessment and monitoring of such trends should dictate screening recommendations, especially in light of new therapies that provide high cure rates and can lower population risk of transmission.

Effective HCV screening programs may or may not be co-located with evaluation and treatment programs. As primary care providers (PCPs) or generalists in a variety of settings begin to treat HCV more often, the barrier of linkage to care (LTC) may be significantly decreased for some populations [5, 6•]. While this model may improve LTC, it may not be feasible in certain regions. HCV clinical programs that do not regularly care for patients prior to diagnosis may disseminate recommendations regarding HCV screening to potential community or healthcare partners along with information to help facilitate effective LTC. Promoting knowledge and screening within the community may enhance the visibility and efficacy of such HCV clinical programs.

## Linkage and access to care

Following diagnosis of active HCV, all patients should be linked to a practitioner able to provide comprehensive HCV management including liver disease severity assessment and HCV treatment [1•]. Patients with advanced fibrosis (Metavir stage ≥ 3) may benefit from consultation with a hepatologist for ongoing advanced fibrosis care to ensure appropriate hepatic health monitoring and screening, and to evaluate for possible liver transplant eligibility [1•].

Improving outcomes for patients with HCV involves creating convenient, accessible, and multidisciplinary methods of care delivery. A number of studies have shown significant divergence between diagnosis and LTC [7–9, 10•]. This breakdown in the cascade of care has become more pronounced with increasing HCV infection among those with active IDU. People who inject drugs (PWID) often face social stigma and unique health system barriers that result in poor HCV care engagement [11]. In one study of 861 suburban heroin users aged 17–35 years in New Jersey, 237 had a positive HCV antibody on screening, but only 16 (6.8%) patients attended an in-office visit, and only 3 (1.3%) initiated DAA therapy. [12]

Dedicating resources for support staff to address LTC, such as a LTC specialist (LTCS) or patient navigator, has shown encouraging results. In one study of methadone clinics, sober living homes, and drug rehabilitation centers, integrating a LTCS resulted in nearly a third (*n* = 116, 29.1%) of HCV patients attending an initial clinic visit [13]. Use of a patient portal for education and appointment information also served to enhance patient experience and engagement in this program [13]. Within a Baltimore sexually transmitted infection (STI) clinic, utilization of a LTC coordinator led to 52% (*n* = 81) of HCV-positive individuals attending an offsite HCV specialist appointment [14]. From these and other LTC studies, it is clear that identification and designation of resources to facilitate LTC from the time of diagnosis can improve the rate at which those with diagnosed HCV complete medical evaluations [11, 15, 16].

In the past, HCV treatment often required referral to a specialist for treatment as well as close monitoring of dose-limiting adverse effects [17•]. In the DAA era, therapy that is safe, effective, and requires minimal monitoring can be delivered effectively by non-specialists and other providers with comparable outcomes [6•, 18, 19]. Co-localizing HCV treatment in settings in which patients are already engaged in care (such as primary care clinics, substance abuse treatment centers, and methadone maintenance facilities) can overcome access barriers for patients unwilling or unable to seek specialty care [20, 21, 22••]. In addition to task shifting, there has been increased emphasis on HCV treatment delivered by PCPs [23]. PCPs are often the first line of patient engagement into the healthcare system and are the most likely specialist to have the opportunity to screen patients for HCV [24]. However, a survey of 80 PCPs found that the majority (70%) did not feel up to date on current HCV treatment options and most severely underestimated the efficacy of DAA regimens [25]. Additionally, one claims-based study from 2010 to 2016 found that only 13.3% of patients with a positive HCV antibody screened by a PCP received treatment [24]. Patients were even less likely to receive treatment if screened by an obstetrician-gynecologist (OBGYN) compared to a PCP (OR 0.493, 95% Wald Confidence Limits 0.353–0.688), with only 4.7% of patients screened by an OBGYN receiving treatment [24].

These findings underline the importance of educating PCPs and non-HCV specialists that are more likely to encounter undiagnosed and untreated patients on the benefits of treatment and treatment algorithms. While task shifting has become a viable option for HCV care delivery, available evidence to support this model consistently involves education of providers that will be implementing new HCV clinical programs [6•, 19]. There are a number of educational resources for providers including the comprehensive University of Washington and University of Alabama at Birmingham “Hepatitis C Online” training course funded by a grant from the Centers for Disease Control and Prevention as well as a variety of other sources (Table 2) [26].

**Table 2:** Practice resources

Practice element	Resource	Description
Provider support	Hepatitis C online course: https://www.hepatitisc.uw.edu/	Comprehensive course including HCV disease state, treatment, and provider resources (e.g., calculators, clinical trial information, landmark study reviews).
	Fundamentals of liver disease: https://www.aasld.org/research-awards/fundamentals-liver-disease	CME module for physicians encompassing viral hepatitis training. This is a collaborative effort of American Association for the Study of Liver Disease, ECHO, the American College of Physicians, CDC, and the Department of Veterans Affairs.
	American Liver Foundation Provider Locator: https://hepc.liverfoundation.org/find-a-healthcare-provider/	Patients can locate treating providers by location and specialty.
	HEP Drug Interactions: https://www.hep-druginteractions.org/	An up-to-date, evidence-based drug-drug interaction resource.
	Clinician Consultation Center: http://nccc.ucsf.edu/clinician-consultation/hepatitis-c-management/	Consultative service through the University of California, San Francisco, for providers managing patients with HCV and co-morbidities such as HIV or substance use. Assists with appropriate HCV therapy selection and issues regarding treatment.
Screening and diagnosis	CDC provider and patient information: https://www.cdc.gov/hepatitis/hcv/index.htm	Provider and patient fact sheets to increase awareness, appropriate screening, and diagnosis practice.
Treatment access	National viral hepatitis roundtable: http://nvhr.org/content/provider-resources	Broad coalition providing resources for navigating prior authorization, sample appeal templates, and best practices for HCV treatment assessment and monitoring.
	HCV treatment access: http://hcvtreatmentaccess.org	Resources to help providers improve access to HCV medications, including sample appeal templates, links to patient assistance programs, and clinical resources.
	Patient Access Network Foundation (PANF): https://panfoundation.org	Patient assistance for HCV patients with high deductibles and co-pays.
	Patient Advocate Foundation (PAF): http://www.patientadvocate.org	Patient assistance for HCV patients with high deductibles and co-pays.
	HealthWell Foundation: https://www.healthwellfoundation.org/	Patient assistance for HCV patients with high deductibles and co-pays.
	The Assistance Fund (TAF): https://tafcares.org/	Patient assistance for HCV patients with high deductibles and co-pays.
	Good Days: https://www.mygooddays.org/	Patient assistance for HCV patients with high deductibles and co-pays.
	Industry resources	DAA manufacturers provide co-pay cards to reduce patient cost after insurance approval. Only patients with non-federal insurance plans qualify for these programs. Co-pay cards may be found on the manufacturer website.

*CME* Continuing Medical Education, *HCV* Hepatitis C virus, *ECHO* Extension for Community Healthcare Outcomes, *CDC* Centers for Disease Control and Prevention

Finally, the use of telehealth technologies to engage patients in care offers promising solutions for a growing population of rural patients. Telehealth programs range from educational and consultative resources such as Project ECHO (Extension for Community Healthcare Outcomes), to specialist and non-specialist patient-facing clinics [27, 28, 29••, 30, 31]. Coverage of telehealth services varies by insurance provider and by state but is increasingly reimbursed [32]. Depending on how these services are provided, telehealth limitations may include lower-quality physical assessment, limited laboratory and imaging availability, and lower quality patient-provider relationship [32]. However, incorporating telehealth services, including video calls, telephone calls, and online platforms, will likely increase patient access to treatment and improve engagement in care in future practice.

## Treatment access

Expanding patient access to HCV therapies is essential in achieving improved patient outcomes. Many public and private insurers initially responded to the high costs of HCV medications by placing restrictions on patient eligibility, such as requiring documentation of advanced liver fibrosis, maintaining a period of abstinence from substance use, and limiting the type of prescriber to gastroenterology or infectious disease specialists [33•, 34–36]. This pattern was reflected by a recent large pharmacy network study, demonstrating an overall HCV therapy start rate of only 70% in 2016, with failure to start primarily driven by insurance denials (83%) [34]. Lo Re and colleagues identified the most common reasons for absolute denial of therapies were insufficient information to assess medical need (36%) and lack of medical necessity (35%) [37•]. These data suggest that current processes in applying for treatment through pharmacy benefits managers (PBMs) are challenging and demonstrate the opportunity for improvements in navigating the approval process.

Multidisciplinary models have implemented a variety of strategies, including patient navigators, nurses, social workers, and specialty pharmacies, to overcome barriers and improve patient access [15, 38, 39]. Patient approval rates utilizing a program nurse alongside a patient navigator were 93% as compared to 81–91% demonstrated in several real-world studies [39, 40, 41•, 42]. Other examples of successful models integrate specialty pharmacies directly into the HCV multidisciplinary team; these models have improved medication approval and access rates while decreasing time to therapy initiation and reducing patient financial burden [43, 44]. Specialty pharmacy integration may include embedding a trained pharmacy technician or pharmacist in an affiliated clinic to work independently or alongside a nurse coordinator [43, 44]. When this type of integration is not an option, developing and maintaining a strong relationship with an external or internal specialty pharmacy may provide similar patient access successes [45].

Regardless of the type of model utilized, a knowledgeable, devoted team skilled in navigating the authorization process is critical to patient access. This process typically begins with a benefits investigation (BI) for insured patients, followed by a prior authorization (PA) request and possible appeals, and ends with financial assistance applied to insured patients’ costs. During the BI, determining and utilizing the PBM-preferred pharmacy, as well as an appropriate preferred treatment regimen, reduces unnecessary denials and delays in treatment initiation. When completing a PA request, it is important to provide all requested clinical information and documentation in order to prevent otherwise unnecessary denials. If all the requested information is not available, it is essential to communicate with and assist the patient in obtaining this information prior to PA submission. In the case of an initial PA denial, it is necessary to determine next steps, such as an appeal or peer-to-peer review. During the appeal or peer-to-peer review, it is recommended to provide supporting documentation as well as evidence for clinical necessity utilizing evidence-based literature to support the request. Resources are available for clinicians and staff to assist with this process including sample appeal templates [46, 47]. If the regimen is denied after exhausting all options for insurance approval, an application for patient assistance through the respective manufacturer may be pursued.

In addition to the insurance restrictions and the approval process, other patient access barriers remain, such as lack of insurance or high out-of-pocket patient costs after PBM approval. Those underinsured and without insurance should pursue assistance through the respective manufacturer, as outlined previously for those with absolute insurance denials. For those patients not participating in state or federally funded insurance programs, manufacturer copay cards may be obtained to defray high copay costs. Alternatively, for those patients enrolled in state or federally funded insurance programs, patient foundations exist (i.e., Patient Access Network Foundation, Patient Advocate Foundation, The Assistance Fund, Good Days, and HealthWell Foundation) for qualifying patients with unaffordable copay costs (Table 2).

## Treatment monitoring

Despite improved safety and efficacy of HCV therapies, on-treatment monitoring remains an important part of the cascade of care. Treatment monitoring in the DAA era includes ensuring adherence through therapy completion, identifying and mitigating drug interactions and adverse effects, and assessment of appropriate response to therapy [48•].

The patient navigator, nurse coordinator, and or specialty pharmacy should remain involved in the monitoring phase in order to ensure continuous patient access to treatment, especially during transitions of care (i.e., from outpatient to inpatient). Following initial approval of HCV medication, approval terms (including approval dates and any on-treatment laboratory requirements) should be reviewed and addressed if needed in order to avoid any potential lapses in treatment. Additional prior authorizations required throughout treatment should also be quickly completed, if applicable.

Once DAA therapy is initiated, it is important to systematically assess medication adherence, potential drug-drug interactions utilizing appropriate resources (such as prescribing information and the University of Liverpool Hep Drug Interactions tool), treatment response utilizing laboratory data, and potential adverse events [49•]. Patients should be monitored as per the AASLD/IDSA HCV Guidance recommendations [48•]. It is currently recommended for patients to be assessed for initial treatment response approximately 4 weeks into treatment, as positive viremia at this time indicates the need for reassessment of adherence, possible drug-drug interactions, and additional testing to ensure an undetectable viral load while on treatment [48•]. Patient safety should also be assessed while on treatment, particularly among those treated with ribavirin-containing regimens, decompensated patients, and those at risk for hepatitis B virus reactivation.

Successful and effective monitoring can be achieved utilizing different models. As previously mentioned, due to the improved safety and efficacy of DAAs, non-specialist providers can effectively treat and monitor HCV patients with comparable outcomes following thorough education and training [6•, 18]. Utilization of non-physician providers could also potentially improve patient adherence to on-treatment visits, as suggested by one study in which treatment visit adherence was higher in those patients seen by a nurse practitioner (73.6% [CI, 69.4 to 77.9%]), as compared to a specialist (55.9% [CI 52.6 to 59.3%]) [6•]. Notably, those patients that achieved SVR demonstrated higher adherence to treatment visits as well (65.8 vs. 40.5%) [6•].

Pharmacists may also contribute directly to therapy monitoring either in clinic or via telemedicine. Studies have demonstrated improved adherence in addition to preventing drug-drug interactions and medication errors with pharmacy involvement [50–53]. More than 50% of HCV patients treated in a multidisciplinary model consisting of an infectious diseases physician, HCV nurse, and specialty pharmacist were identified to have drug-drug interactions requiring interventions by a pharmacist [50]. Initial treatment education and follow up monitoring every 4 weeks were completed by the HCV nurse and pharmacist, as well as one on-treatment office visit with the provider [50]. Utilizing this model, no patients stopped treatment due to adverse effects and 79.1% of patients reported 100% adherence rates [50].

Telemedicine has the potential to improve adherence in patients on treatment as well, particularly among those with limited provider access. Although there is limited data available on those patients treated with DAAs, studies have demonstrated similar SVR rates for those telemedicine and non-telemedicine patients treated with DAAs as well as pegylated interferon and ribavirin [54–56]. Additionally, adverse drug events were more likely to cause non-telemedicine patients to discontinue treatment than those telemedicine patients receiving the same treatment [5, 55].

It is important to recognize that patients infected with HCV often have social and psychological barriers to adherence and treatment completion and may require additional support [11]. Some models incorporate mental health professionals and social work into clinical programs, resulting in high HCV treatment completion rates [57]. Strategies to assist patients with complex needs have been described by Sublette et al., including patient advocacy (i.e., housing and income stability during treatment), practical problem solving to help patients adhere, ongoing feedback to provide positive reinforcement, and emotion-focused support to assess psychological impact of treatment and improve mood [58]. Another study more specifically addressed adherence and outcomes by utilizing direct observation of DAA in PWID receiving opioid agonist therapy under direct observation by a nurse or physician. This direct-observation model resulted in high adherence, with 1875 of 1878 scheduled DAA doses ingested, as well as high SVR rates (*n* = 40; 100%), demonstrating that previously difficult-to-treat patients can have successful outcomes [59•].

## Post-treatment engagement

Continued patient engagement in medical care beyond completion of DAA therapy is essential for effective HCV clinical programs. All patients who complete DAA therapy should be evaluated for SVR at least 12 weeks after completing therapy to confirm clinical response and to determine the plan for subsequent liver care [48•].

For those who do not achieve SVR, ongoing disease assessment should occur every 6–12 months and, if appropriate, cirrhosis care including hepatocellular carcinoma monitoring. Recommendations for retreatment after DAA failure with new DAA agents are available, and HCV clinical programs should be prepared for a small number of patients to relapse despite appropriate treatment and treatment adherence [60•]. As multiple DAA therapy options are now recommended by the Food and Drug Administration for retreatment after DAA failure, retreatment strategies have become more feasible for clinical programs.

For those who achieve SVR after DAA treatment, the need for ongoing medical care is determined by the degree of liver fibrosis present. Patients who do not have advanced fibrosis and who achieve SVR do not require further liver care and monitoring; these patients may follow-up “as if they were never infected with HCV” as per the AASLD/IDSA guidance. Patients who achieve SVR but have cirrhosis need ongoing liver care including hepatocellular carcinoma monitoring at appropriate intervals as well as baseline endoscopy as part of the HCV clinical program or after referral for further care [48•].

All patients who achieve SVR require counseling. These patients should be aware that screening tests based on HCV antibodies will remain positive lifelong despite SVR. Patients who achieve SVR should be educated that engaging in high-risk behaviors may result in HCV reinfection. Reinfection rates have varied greatly across cohorts depending on risk behaviors observed [61•, 62–64]. High-risk groups, including PWID, men who have sex with men, and those with HIV/HCV coinfection, should receive specific education including risk reduction strategies. HCV clinical programs should be able to provide HCV reinfection screening for patients who remain at risk or educate patients regarding screening resources available in their community.

## Case example

**Fig. 1:**
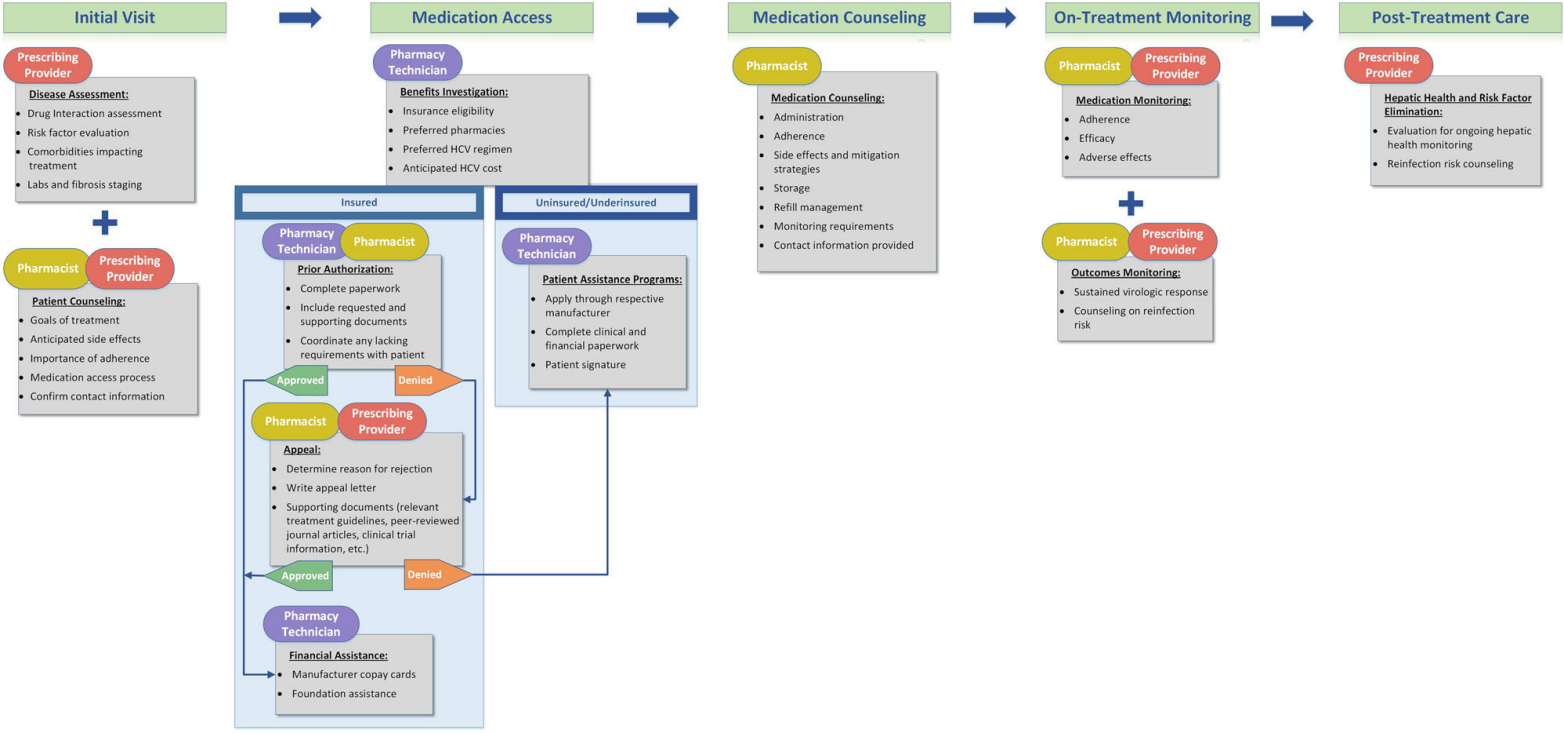
Vanderbilt University Medical Center Division of Gastroenterology, Hepatology, and Nutrition and Division of Infectious Diseases employ a multidisciplinary approach in hepatitis C care delivery that integrates a prescribing provider (physician, nurse practitioner, or physician’s assistant), a pharmacist, and a pharmacy technician into clinical practice. The figure describes the cascade of care and corresponding responsibilities from an initial clinic visit through treatment completion and sustained virologic response.

As a large academic health system with an integrated specialty pharmacy, Vanderbilt University Medical Center (VUMC) offers a singular example of practices to improve clinical outcomes. Patients referred to VUMC for HCV care are treated either through the Division of Gastroenterology, Hepatology, and Nutrition (GI) or through the Division of Infectious Diseases (ID). Within both of these clinics, specialty pharmacist(s) are embedded that serve as medication experts but also effectively as patient care coordinators, ensuring that patients are able to efficiently navigate from an initial visit to the anticipated clinical outcome of SVR. This process is detailed in Fig. 1.

Multidisciplinary care begins with a patient’s initial visit to one of the VUMC outpatient clinics. Following a disease assessment by a prescribing provider (physician, physician’s assistant, or advanced practice nurse practitioner), the patient is contacted by a pharmacist either within the same clinic visit (ID) or by phone subsequently (GI). The pharmacist counsels the patient on the process of obtaining treatment and provides preliminary medication education. The pharmacist ensures necessary laboratory, staging, and supplemental insurance requirements (e.g., resistance testing, rehabilitation certificates, adherence assessments) are completed either at the initial visit or as early as possible. A comprehensive medication reconciliation is performed and any possible drug interactions with DAA therapy are addressed. This early relationship between patient and pharmacist provides patients an accessible care coordinator, reducing patients’ risk for being lost to follow-up after an initial visit. For other practices unable to integrate clinical pharmacists, prescribing providers could address potential drug-drug interactions, and clinic staff such as nurse coordinators, social workers, or case managers could play the vital role of educating patients regarding treatment access and ensuring appropriate work-up completion.

Within the VUMC model, the most appropriate treatment is collaboratively agreed upon by the pharmacist and prescribing provider after completion of disease state assessment. Coordinating medication access is a key role of both the integrated pharmacist(s) and pharmacy technician (Fig. 1). The integrated model of care is advantageous as it allows pharmacists full access to necessary medical information that is required for treatment access document completion. Additionally, forms requiring a patient signature can be easily completed at an initial visit if the pharmacist identifies insurance-specific or patient assistance program (PAP) requirements. Pharmacists and technicians complete PAs and appeals as necessary for patients with insurance. When patients are denied treatment after multiple appeals (underinsured), the pharmacist attempts to obtain treatment through manufacturer programs. Within the ID clinic over 14 months, this model resulted in a 96% medication access rate, with only 3% of people denied treatment, all with Medicaid insurance [53]. Again, in clinical programs where these integrated pharmacy services are not available, developing a partnership with a specialty pharmacy that frequently assists with DAA treatment may be helpful. Upon practice establishment, providers should recognize specific clinic staff (e.g., nurses, technicians) that specialize in helping patients access treatment and can become familiar with the process and the requirements.

Following treatment approval, pharmacists educate patients on the importance of adherence to therapy, what to expect while on therapy, and necessary monitoring on and after treatment. Patients receive direct contact information to facilitate treatment adherence if troublesome side effects and/or other concerns arise. This role is best fulfilled by a pharmacist, though if a pharmacist is not integrated into a clinical program or able to document patient understanding in the electronic medical record, clinic staff should confirm patients' understanding of the medication education provided by the dispensing pharmacy.

At clinic follow-up visits, prescribing providers complete necessary lab monitoring and provide further counseling on treatment plans and post-treatment requirements. In the interim, pharmacists monitor patient side effects, adherence, and for any possible lapses in therapy due to hospitalization, incarceration, or other situations that may arise. The pharmacy technician calls patients 7–10 days prior to completion of their first month of therapy to ensure timely medication delivery and clinic follow-up. These roles could be fulfilled by nursing coordinators or others for clinical programs without integrated pharmacy staff.

Once treatment is completed, patients are assessed for an SVR at least 12 weeks following treatment completion. If an SVR is achieved, the prescribing provider will discuss the need for ongoing liver care and hepatocellular carcinoma monitoring and counsel patients regarding future care as described above. In the infrequent instance when a cure was not achieved, patients are evaluated for further treatment.

Within this model of care, over 1500 patients have been treated and cured of HCV over the last 5 years. The successful outcomes of this model are due in part to a multidisciplinary team focused on patient engagement, education, and empowerment.

## Conclusion

The impact of HCV therapy on population health hinges on increasing screening and treatment as well as reducing HCV exposure risk. Successful strategies to identify and treat patients with HCV should include frontline providers such as PCPs, and clinical programs should employ a multidisciplinary approach to effectively engage and retain patients through treatment completion. Finally, ensuring response to treatment and advocating post-treatment monitoring as well as HCV risk reduction is essential to promote optimal HCV treatment outcomes. With effective HCV treatment now the standard of care, ongoing research should focus on improving identification of patients with active infection, best practices in delivering HCV care, and innovative strategies to reduce and eliminate the spread of HCV.
